# Modeling RNA secondary structure folding ensembles using SHAPE mapping data

**DOI:** 10.1093/nar/gkx1057

**Published:** 2017-11-21

**Authors:** Aleksandar Spasic, Sarah M Assmann, Philip C Bevilacqua, David H Mathews

**Affiliations:** Department of Biochemistry & Biophysics, University of Rochester Medical Center, Rochester, NY 14642, USA; Center for RNA Biology, University of Rochester Medical Center, Rochester, NY 14642, USA; Department of Biology, Pennsylvania State University, University Park, PA 16802, USA; Department of Chemistry, Department of Biochemistry & Molecular Biology, Center for RNA Molecular Biology, Pennsylvania State University, University Park, PA 16802, USA; Department of Biostatistics & Computational Biology, University of Rochester Medical Center, Rochester, NY 14642, USA

## Abstract

RNA secondary structure prediction is widely used for developing hypotheses about the structures of RNA sequences, and structure can provide insight about RNA function. The accuracy of structure prediction is known to be improved using experimental mapping data that provide information about the pairing status of single nucleotides, and these data can now be acquired for whole transcriptomes using high-throughput sequencing. Prior methods for using these experimental data focused on predicting structures for sequences assuming that they populate a single structure. Most RNAs populate multiple structures, however, where the ensemble of strands populates structures with different sets of canonical base pairs. The focus on modeling single structures has been a bottleneck for accurately modeling RNA structure. In this work, we introduce Rsample, an algorithm for using experimental data to predict more than one RNA structure for sequences that populate multiple structures at equilibrium. We demonstrate, using SHAPE mapping data, that we can accurately model RNA sequences that populate multiple structures, including the relative probabilities of those structures. This program is freely available as part of the RNAstructure software package.

## INTRODUCTION

RNA has many important roles in the cell besides being a simple carrier of genetic information (1). RNA sequences are an essential part of translation through tRNA and rRNAs (2), they play a role in regulating gene expression through microRNAs (miRNA) and small interfering RNAs (siRNAs) (3), and in RNA processing through small nuclear RNAs (snRNA) (4). In addition, many RNAs require conformational change in response to a stimulus to function, including riboswitches, ribozymes and protein-complexed RNAs (5–7). Knowledge of RNA structure is key in understanding the intermolecular interactions of small RNAs with their mRNA targets, as well as in understanding RNA-protein interactions. In addition, changes to RNA structure as a result of sequence variants can lead to human disease (8).

RNA structure can be mapped experimentally at the level of individual nucleotides using enzymatic or chemical methods (9,10). Many insights regarding RNA structure have resulted because new mapping methods have been developed, and because of new methods that quantify the extent of reactivity to agents (11–13). One newer chemical mapping method is SHAPE, which covalently modifies the sugar 2′ oxygen of nucleotides that are in flexible regions of the structure (14). Recent work focused on acquiring these experimental mapping data for entire transcriptomes (15–17) and also on acquiring these data *in vivo* (18–20). While these and other chemical mapping data provide information about the likelihood that a nucleotide is base paired, they do not directly provide the structure of the RNA. To model the structure, the mapping data have been used to restrain secondary structure prediction. These structural models provide important insights into RNA structure and function.

Several methods are available to model structures using mapping data (21–27). A common weakness, however, is that the leading methods for using mapping data assume a single conformation for the RNA (21,28,29). However, many sequences, including mRNA, riboswitches, and long-non-coding RNA (30), are expected to populate a variety of structures *in vivo*. Other computational methods have attempted to model multiple structures at equilibrium using SHAPE data (31,32). These approaches assume that the extent of reactivity at a given nucleotide indicates the fraction of molecules in which that nucleotide is unpaired at equilibrium (31–33). Experiments show a variety of reactivities depending on local structure, however, and nucleotides that are base paired in ribosomal RNA can be extensively reactive (34). Thus these approaches might misinterpret a considerable fraction of the experimental data. Given the current lack of tools to model sequences that populate multiple conformations using experimental restraints, analysis of transcriptome-wide structure has often focused on direct observations about reactivity data, rather than relying on models of the structures (18,20). This lack of computational tools is a bottleneck to using transcriptome-wide data to learn about the details of RNA structure and how it changes in response to physiological challenges.

Here, we introduce the algorithm Rsample (for restrained sample), which models RNA secondary structure using thermodynamics guided by structure mapping data. Rsample addresses two limitations in prior methods. First, it explicitly considers that multiple copies of the same sequence can simultaneously fold to different structures. Second, it focuses on the agreement between experimental mapping data and estimated mapping data by sampling RNA structure models, rather than interpreting data in the absence of structure models. This technique provides a principled approach for integrating thermodynamic prediction with mapping data.

We tested Rsample for accuracy at predicting structure and populations of individual conformation using SHAPE mapping data. For sequences that fold into multiple conformations, our equilibrium predictions are generally accurate within a factor of about 2*kT*, where *k* is the Boltzmann constant, *T* is the absolute temperature (310 K), and *kT* represents the conformations that would be accessible to an RNA because of thermal fluctuations. We also benchmarked Rsample for sequences that populate a single structure, and its performance was similar to other methods that can use SHAPE data: RME (29), RNAprob (28), RNAprobing (31), RNAsc (32) and RNAstructure Fold (21).

## MATERIALS AND METHODS

### Algorithm

**Figure 1.**
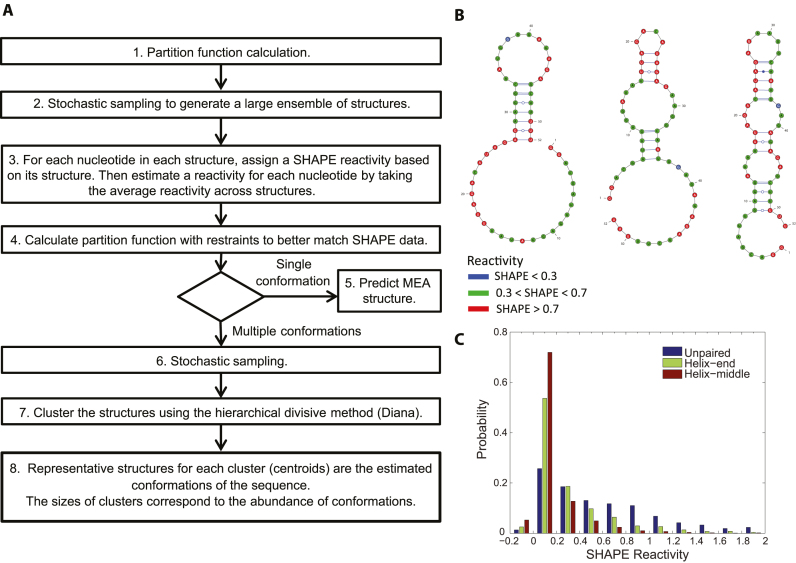
Overview of Rsample and experimental data. (**A**) The Rsample procedure. (**B**) The three conformations of a designed FMN riboswitch, color coded with SHAPE reactivity from Cordero *et al.* (43). The observed SHAPE reactivities are the weighted means of the reactivity of each structure. In the natural mixture in solution, the reactivity of each structure cannot be observed. (**C**) Distribution of normalized SHAPE reactivities for unpaired nucleotides, nucleotides paired at helix ends, and nucleotides paired and not at helix ends. The distributions show significant overlap and peak at a reactivity of 0, although the distribution for unpaired nucleotides skews farther towards higher reactivities. Nucleotides at the ends of helices also have some skew to higher reactivities than nucleotides in the interior of helices.

An overview of Rsample is shown in Figure 1a. The procedure uses two partition function calculations to model structure (23,35). A partition function calculates the probability of a structure using estimates of folding thermodynamics and can also calculate the base pairing probability for each possible base pair. The first partition function calculation (Step 1) establishes how well the various structures predicted by the pure thermodynamic model (i.e. without any restraints from experiments) estimate the experimental reactivities for each nucleotide in the sequence. The second partition (Step 4) function re-estimates the probabilities of structures to better match the experimental data by introducing restraints at the nucleotide level.

Steps 1 and 2 generate an ensemble of structures using a thermodynamic model for RNA folding (24). The partition function (step 1) and stochastic sampling (36) (step 2) generate a set of 10 000 (possibly non-unique) structures, where the probability of sampling a structure is the Boltzmann probability of the structure occurring at equilibrium. Ding and Lawrence (36) found that two independent 1000 conformation samples of an 1187 nt long sequence had an almost identical probabilities of nucleotides being paired. In addition, we found that using more than 10 000 conformations (from step 2) did not change the predicted normalized reactivities by >0.01, and had no effect on predicted structures. Therefore a sample of 10 000 structures was used for stochastic sampling.

In step 3, the SHAPE reactivity for each nucleotide is estimated based on the structure ensemble ($Rcalc$). Reactivity estimates are generated for each nucleotide in each structure as described below, and the experimentally observed reactivity is assumed to be a population-weighted average of the reactivity of each structure. The fact that the observed reactivity is a population-weighted average obfuscates the reactivity for any given unique structure present in a mixture of different structures; for example, no single structure from the folding ensemble of the flavin mononucleotide-binding (FMN) riboswitch (Figure 1B) is consistent with the experimental reactivity pattern observed. To assign an estimated reactivity for a nucleotide, the distributions of SHAPE reactivities observed for structured RNAs (Figure 1C) is called upon. Prior work demonstrated that in addition to paired nucleotides, unpaired nucleotides can be unreactive to SHAPE (34), probably because base-base interactions (hydrogen bonds and stacks) can limit the motion of unpaired nucleotides in loops. We use three reactivity distributions: reactivities for those nucleotides that are unpaired (Figure 1C, blue), those at helix ends (Figure 1C, green), and those in helix interiors (Figure 1C, brown). A nucleotide in a structure receives a reactivity from the proper reactivity distribution drawn at random, but weighted by the distribution. The total estimated reactivity for a nucleotide is the arithmetic mean for that nucleotide across all sampled structures.

In step 4, in order to revise the structure predictions so that the estimated SHAPE reactivity better matches the experimentally observed reactivity, the estimated SHAPE reactivities are used to calculate a pseudo-free-energy change:

(1) \begin{equation*}\ \Delta {G_{bonus,\ i}} = \ C \times \ln \left( {\frac{{Rex{p_i} + Offset}}{{Rcal{c_i} + Offset}}} \right)\end{equation*}

where$\ Rex{p_i}$ and $Rcal{c_i}$ are experimentally measured reactivities and estimated reactivities, respectively, of nucleotide *i*. $C$ and $Offset$ are parameters that have been optimized by finding the most accurately predicted structures over many values of parameters. $C$ has units of energy (kcal/mol) and is used to establish the relationship between free energy and reactivities. The parameter $Offset$ is unitless and it is introduced to accommodate the fact that normalized reactivities can be equal or less than zero.

$\Delta {G_{bonus,\ i}}$ is then incorporated into the partition function calculation (step 4) as a restraint. As in our prior work, $\Delta {G_{bonus,\ i}}$ is added to the folding free energy of base pair stacks for each nucleotide in the stack (21). Nucleotides interior to helices receive this free energy change twice because they are in two base pair stacks. Nucleotides at helix ends receive this free energy bonus once. In this re-estimation of the partition function, nucleotides with estimated SHAPE reactivity that is lower or higher than that observed by experiment are restrained, for the first time, with a lower or higher propensity to base pair, respectively, than in the first partition function calculation. Nucleotides with estimated SHAPE reactivities that match the experiment receive no restraint because the thermodynamic prediction already matches the experiment for those nucleotides.

At this point in the procedure, the modeling can proceed for either modeling multiple structures or a single structure. If a single structure is either suggested by the presence of a single cluster of highly similar structures or is strongly expected from other physicochemical/biological criterion, the partition function can be used to predict the maximum expected accuracy structure (37,38) (Step 5). When multiple structures are suggested by clusters of different structures or more than one structure is expected, the procedure continues to steps 6, 7 and 8. Step 6 samples 1000 structures from the Boltzmann ensemble, and this sample is then clustered in step 7. A cluster is defined as a subset of structures with similar base pairs. The optimal number of clusters is determined automatically using the Calinski-Harabasz (CH) index, which attempts to choose the minimum number of structures that adequately reflects diverse folds (39). For a given cluster, the probability of base pairing for each pair of nucleotides is determined as the probability of the pair appearing in a structure in the cluster. In step 8, centroid structures are assembled to include all pairs with pairing probability >0.5. Centroids of clusters then represent conformations with the population determined by the cluster size.

Rsample is programmed in C++ and is incorporated in our software package RNAstructure (40). Each secondary structure prediction step (1,2,4,5,6) is implemented using the RNAstructure class library. This partition function does not include pseudoknots, and therefore pseudoknots are not predicted. The computational time of Rsample is about twice our previous method because two partition function calculations are performed. Although many RNA sequences are known to have pseudoknots, the number of base pairs in pseudoknots is generally small compared to the total number of base pairs (41). This limitation in secondary structure prediction is common (42); possible pseudoknots can be identified using base pairing probability estimates (41).

### Database of structures and reactivities

A collection of structures with measured SHAPE reactivities and known secondary structure was used to determine distributions of unpaired nucleotides, paired nucleotides at the end of helices, and paired nucleotides not at the terminal positions of helices (Figure 1c), to determine model parameters (*C* and $Offset$), and to test the performance of the methods for predicting secondary structure of single conformation sequences (Figure 3). The 16 sequences used here are from work of Hajdin *et al* (22). For the riboswitches in these data, mapping was performed under conditions where one structure is populated. The sequences range in length from 34 to 2904 nucleotides for a total of 6514 nucleotides with measured SHAPE reactivity. The full list of sequences is given in Table S1 in the Supplementary Data.

Two sources were used for sequences known to have multiple conformations and for which SHAPE reactivities have been measured. First, data was collected for the 5 sequences from Cordero *et al.* (43) under conditions where multiple structures were present. The ratio of conformations was determined using the procedure developed to interpret mutate-and-map experiments and compared to experimental data if available. In our calculations we use the experimental data for the ratio if they are available. The second source of data was the HIV-1 Rev response element (RRE) sequence where the two conformations had their SHAPE reactivities measured independently (44). We then estimated the equal mixture of the two conformations by calculating the mean of reactivities. The list of sequences used is given in Table 1.

**Table 1.** List of benchmark sequences known to fold to multiple conformations

	Name	Length	Conformational ratio	Source for conformation ratio
**1**	HIV-1 RRE	232	50% + 50%	Computed here from SHAPE data measured for separate conformations (44)
**2**	Bistable Sequence	25	70 ± 5% + 30 ± 5%	NMR data (47)
**3**	16S rRNA Four way Junction	110	90% + 10%	Estimate from Mutate-and-Map (43)
**4**	ADD Riboswitch, *V. Vulnificus*	112	(–ADD): apoA: 32% + apoB: 68%	NMR data and Estimate from Mutate-and-Map (43,48)
			(+ADD): holo: 70% + apoA + apoB: 30%	
**5**	MedLoop	35	96% + 4±4%	Designed sequence, Estimate from Mutate-and-Map (43)
**6**	FMN Riboswitch	52	(–FMN): A: 56 ± 16% +B: 27 ± 12% + C: 17 ± 11%	Designed sequence, Estimate from Mutate-and-Map (43)
			(+FMN):A: 40 ± 8% + B: 50 ± 10% + C: 10 ± 5%	

The ‘conformational ratio’ is the percent population of each known conformation. The two riboswitches have data recorded with (+) and without (–) the conformation-changing ligand.

### Benchmarks and measures of accuracy

The ‘No SHAPE’ control method was stochastic sampling (36), followed by clustering (39). This represents steps 1, 6, 7 and 8 from the flowchart in Figure 1A, and it the natural comparison for the absence of data. Benchmarking of all methods was done using default settings.

Accuracy of prediction was determined by calculating sensitivity and positive predictive value (PPV). Sensitivity measures the percentage of known base pairs that occur in the predicted structure and PPV is the percentage of predicted pairs that occurs in the known structure. A base pair (*i* to *j*) was considered correctly predicted if the comparison contains the pair (*i* to *j*), (*i* + 1 to *j*), (*i* – 1 to j), (*i* to *j* + 1) or (*i* to *j* – 1). This is important because the accepted structures cannot distinguish these possibilities and because thermodynamic fluctuations can make it possible to sample these alternative structures.

To test the statistical significance of benchmarks of methods for predicting single conformation, a two-tailed, paired *t*-test with type I error rate set to 0.05 was performed (45). The null hypothesis is that two methods perform with equal accuracy on the benchmark. The p values were calculated with Microsoft Excel 2010.

### Determination of parameters $C$ and $Offset$

Rsample requires determination of two parameters, $C$ and $Offset$ (equ. 1). They were determined from the optimal parameters found using a two dimensional grid search across the 225 combinations of values of parameters starting from 0.1 and ending at 1.5 and plotting the average accuracy. The resulting two-dimensional plot of geometric mean of sensitivity and PPV for all sequences from Supplementary Table S2 is given in Supplementary Figure S2. The maximum is a value of 82.8% at $C$= 0.5 kcal/mol and $Offset$= 1.1, but we note that there is a large triangular area where the geometric mean has values of 80% or more. The robustness of fit is tested by the jackknife method where a set of $C$ and $Offset$ parameters was derived for all subsets of sequences in database where one sequence was left out. We found that each subset produced the same set of parameters.

### Shannon entropy

The mean Shannon entropy, *S*, per nucleotide can be used to estimate the ensemble diversity in base pairing. This is estimated using (23,46):

(2) \begin{equation*}S\ = \ - \frac{{\mathop \sum \nolimits_{i,\ j} {P_{i,\ j}}\log \left( {{P_{i,\ j}}} \right)}}{N}\end{equation*}

where *P_i, j_* is the probability of the base pair between nucleotides i and j, *N* is the sequence length, the log is base 10, and the sum is taken over all possible canonical base pairs.

## RESULTS

### Modeling RNA sequences with known multiple conformations

**Figure 2.**
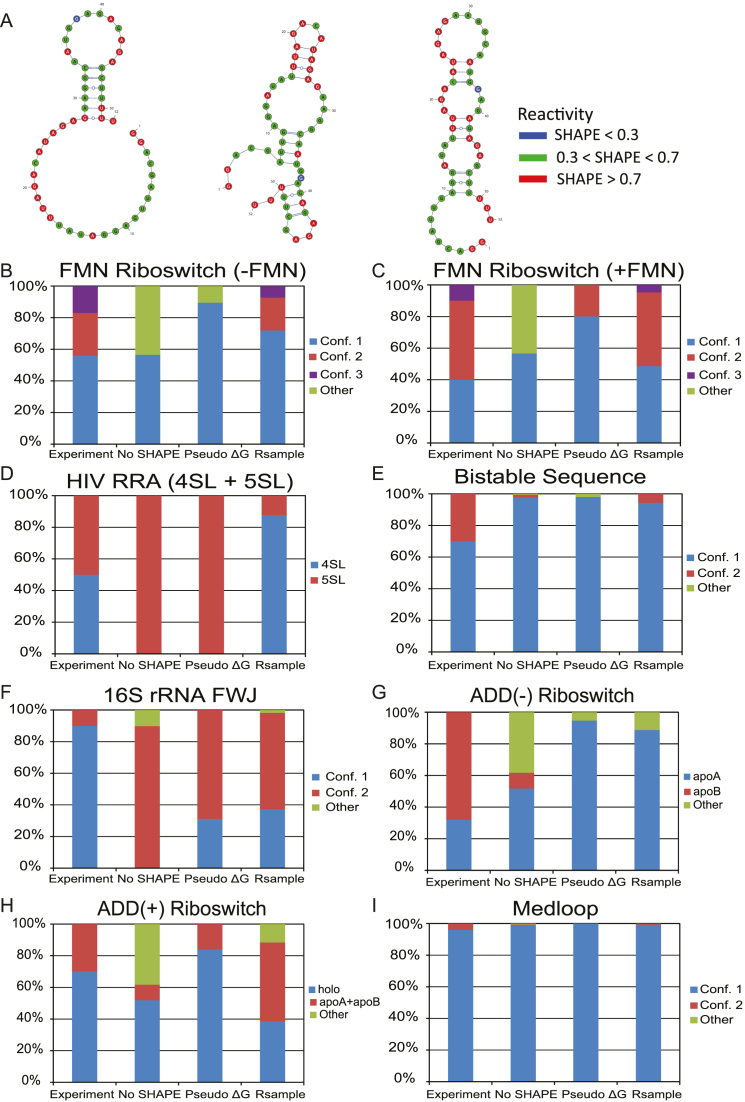
Performance of methods for predicting the ratio of known conformations. Experimental ratios are compared to the predicted ratios for the method without using SHAPE restraints (No SHAPE), using our original method for including restraints (Pseudo ΔG) (21), and the method introduced here (Rsample). (**A**) The predicted centroid structures for the FMN riboswitch in the absence of ligand using Rsample. The three accepted structures are shown in Figure 1B. The remaining plots show the models for each sequence, FMN riboswitch (–FMN; **B**), FMN riboswitch (+FMN, **C**), HIV RRA (**D**), bistable sequence (**E**), 16S rRNA (**F**), ADD riboswitch (-adenine; **G**), ADD riboswitch (+adenine; **H**), and medloop (**I**). To illustrate the performance of the whole predicted ensemble predicted ratio for structures that were not close to any of the known conformations (‘other’) is also given.

From the literature, we assembled a database of 6 sequences that are known from experiments to fold into two or three structures and for which SHAPE mapping data are available (Table 1) (43,44,47,48). For two of the sequences, the adenosine-binding riboswitch upstream of adenosine deaminase (ADD riboswitch) and FMN riboswitch, experimental mapping data are available separately for the presence or absence of ligand. These switches are each known to fold into three total structures, with different populations depending on whether ligand is bound or not, and the populations are known. We benchmarked Rsample, our prior method (pseudo ΔG with SHAPE restraints (21)), and stochastic sampling without SHAPE restraints ‘No SHAPE’, which is equivalent to Rsample without using SHAPE-directed refinement. In addition, Supplementary Figure S3 shows the predictions of RNAprobing (31) and RNAsc (32), which calculate partition functions and are therefore able to generate structure samples. Two measures were used to compare the predictions with experimental data. First, the accuracy of structure modeling is assessed by comparing the centroids, i.e. our structure models, to all experimentally known structures. Second, by examining the sizes of clusters from which the centroids were calculated, the estimated population of each conformation is compared to the experimentally known ratio of conformations. Table 2 and Figure 2 report the findings of these two measurements by comparing to experimental data for Rsample, Pseudo ΔG and No SHAPE, while Supplementary Table S3 and Figure S3 report these measurements for RNAprobing and RNAsc. Accuracy is characterized as the geometric mean of sensitivity and positive predictive value. Sensitivity is the fraction of known base pairs that appear in the centroid and positive predictive value is the fraction of base pairs in the centroid that appear in the known structure. Therefore, when pairs are missing from a centroid, the sensitivity is lowered; when incorrect pairs are predicted, the positive predictive value is lowered.

**Table 2.** Accuracy of stochastic sampling without SHAPE data (No SHAPE), our prior method (Pseudo ΔG) (21), and Rsample (36) for predicting the structures of sequences with multiple conformations

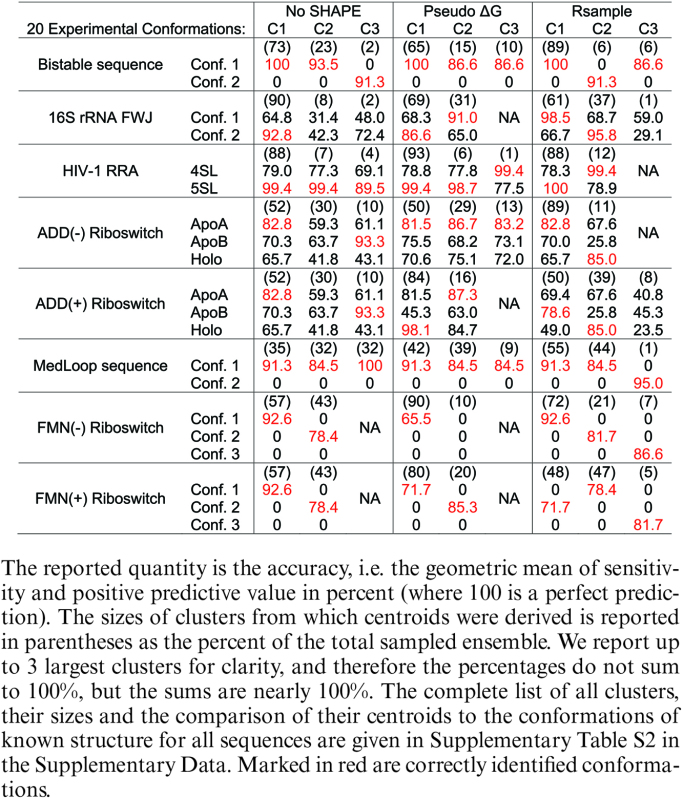

We begin by considering the accuracy of structure modeling different conformations. Table 2 and Supplementary Table S3 report accuracy of centroids of the three largest clusters (‘C1’, ‘C2’, and ‘C3’) compared to the two or three experimentally verified conformations (in rows) for each sequence. We consider that Rsample correctly identified a conformation if the accuracy is higher than 80% and the identified structure is not closer to any other known conformation. In the case of the FMN riboswitch, we lower the accuracy criterion to 70% considering that it is an artificially designed sequence (49), i.e. it is not known in biology, and its secondary structure has not been independently verified. Out of the total of 20 experimentally known conformations for the six sequences, using no SHAPE restraints identifies only 13 conformations correctly, our prior method with SHAPE restraints, ‘Pseudo ΔG’, identifies just 12 correctly, but Rsample identifies a remarkable 18 conformations correctly. RNAsc (32) and RNAprobing (31) correctly identify 12 and 13 conformations respectively (Supplementary Table S3), thus performing similarly to our prior method with the Pseudo ΔG SHAPE restraints (21).

We next consider populations of the different conformations. Figures 2 and Supplementary Figure S3 compare the population ratios of conformations predicted by Rsample and other methods to those measured by experiment. In normal use, a user would not know how many conformations should be expected, so in addition to the populations of known conformations, we report the fraction of conformations that were not close to known conformations. Also, the clustering procedure can produce multiple clusters whose centroids predict the same known secondary structure with at least 80% accuracy. We consider such clusters to represent the same conformation, and allow a centroid to predict one single known conformation to which it is closest in structure. The full list of centroids, the sizes of their clusters and their proximity to known conformations are given in Table S2 in the Supplementary Data.

Figures 2 show that Rsample is able to estimate multiple clusters, each of which correctly correspond to an experimentally known conformation. For example, the FMN riboswitch without ligand populates three experimentally known structures at equilibrium, and Rsample is able to reproduce these three structures. Additionally, the relative abundance of each structure is correctly predicted. The relative abundance of two known structures is also correctly predicted for the bistable sequence, ADD riboswitch bound to adenosine, and MedLoop. For HIV RRA, 16S rRNA FWJ, and the FMN riboswitch with ligand, the known structures are populated, but the relative abundance is incorrectly predicted. By contrast, the performance of our prior method (21) with SHAPE restraints (Pseudo ΔG) to model multiple conformations is poor. Specifically, while our prior method is sometimes able to identify multiple conformations, it generally predicts an ensemble consisting primarily of one conformation, as evidenced in Bistable sequence, HIV RRA, ADD Riboswitch, and FMN riboswitch without ligand. This highlights the importance of correctly modeling of the populations of multiple conformations. The prediction using sampling but without SHAPE data (36) also tends to predict one dominating conformation, and it often also predicts structures that are not close to any of the known conformations. This highlights the importance of experimental mapping data for structure prediction accuracy. The performances of RNAsc (32) and RNAprobing (31) for identifying the ratio of conformations are shown in Supplementary Figure S3. RNAprobing correctly predicted ratios of conformations for the Bistable sequence and HIV RRA. RNAprobing, however, estimated incorrect structures for a majority of the conformational populations for the ADD(- ligand) and FMN(- ligand) riboswitches. RNAsc correctly predicted the ratios for the 16S rRNA FWJ and ADD(+ ligand) riboswitch, but overstabilized a single structure for the Bistable Sequence ADD(- ligand) riboswitch, and Medloop.

### Shannon entropy

Shannon entropy has been used in conjunction with SHAPE data to identify regions of sequences could participate in formation of multiple conformations or do not participate in a specific structure, i.e. regions of sequence that form a wide variety of base pairs in the thermodynamic ensemble that have little functional significance (46,50,51). Supplementary Figure S4 in the Supplementary Data compares mean Shannon entropies calculated on the dataset of sequences with multiple conformations (Table 1) using Rsample and our earlier pseudo ΔG method for including SHAPE restraints. Averaged over all sequences, Rsample predicts higher Shannon entropy per nucleotide (0.19 versus 0.07) indicating Rsample predicts structures with more flexible base pairing, which suggests that explicitly modeling multiple conformations provides better estimates of structural variability. This is expected given the better modeling of clusters using Rsample as compared to using the prior pseudo ΔG model (Table 2 and Supplementary Table S2).

### Benchmark for single conformation sequences

**Figure 3.**
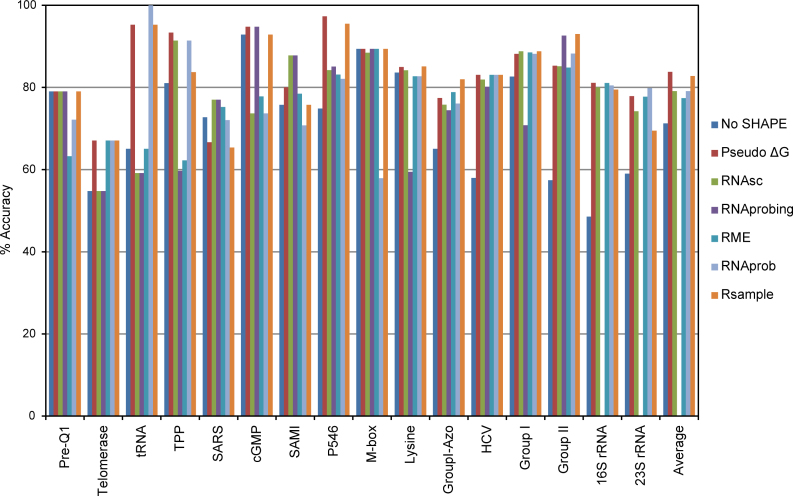
Performance of our method (Rsample) compared to other methods for predicting single conformation. The results are given as geometric mean of sensitivity and positive predictive value. RNAprobing predictions for 16S rRNA, 23S rRNA and its average over all sequences are not included because RNAprobing does not handle sequences longer than 1, 000 nucleotides.

In addition to examining performance for multiple conformations, we tested Rsample for single conformations with the expectation that it would perform well despite its explicit design for modeling multiple structures. Using SHAPE data from the literature (22), we compared Rsample with a number of methods: a control method that does not incorporate SHAPE restraints (No SHAPE), our previous Pseudo ΔG method (21), RNAsc (32), RNAprobing (31), RME (29) and RNAprob (28). The accuracy results are given in Figure 3. The equivalent figures for sensitivity and positive predictive value are given in Supplementary Figure S1 in the Supplementary Data.

The results for individual sequences and the average over all sequences demonstrate that all methods that use experimental restraints perform similarly. All methods perform better when SHAPE data are used. Methods that use experimental restraints produce accuracy of around 80%. Our prior method (Pseudo ΔG) leads with an accuracy of 83.8% and Rsample has essentially the same accuracy at 82.8%. A two-tailed, paired *t*-test showed no statistical significance for the differences in accuracy of Rsample and the other methods that use SHAPE (45). The stochastic sampling method that uses no experimental restraints has an accuracy of 71.2%, indicating a substantial improvement in structure prediction accuracy of about 10 percentage points when using SHAPE restraints compared to not using them for this dataset. Rsample is more accurate than the control method not using SHAPE with statistical significance (*P* < 0.05).

## DISCUSSION

Here we present Rsample, a new algorithm that solves the critical problem of interpreting experimental chemical mapping data transcriptome-wide while generating ensembles of structures for each sequence. Prior work largely focused on using SHAPE, enzymatic, and DMS mapping data to improve the accuracy of structure prediction for structured RNAs by assuming that they have a single predominant structure *in vivo*. Rsample provides a new dimension in interpreting these data. We focused on SHAPE data for this manuscript, but Rsample should be generalizable to other mapping reagents such as DMS (52) and other chemicals, as well as enzymatic mapping data (29).

The mutate-and-map technique, and the software developed for interpreting those data, is also capable of modeling structural ensembles (43). In fact, many of the test cases (Table 1) used here have ensemble populations estimated using mutate-and-map. But mutate-and-map requires experimental mapping data on a large number of sequence mutants, and therefore mutate-and-map is not able to model RNA structures transcriptome-wide and *in vivo* mapping by mutate-and-map would be challenging, and is not currently possible. Rsample, however, is capable of inferring ensembles of structures using the mapping data on a single sequence, and therefore it could be applied transcriptome-wide and using *in vivo* data.

Rsample uses a principled approach to restrain structure prediction. The focus is on generating structure ensembles with estimated SHAPE reactivities that match the experimentally measured reactivities. The number of distinct clusters is small for the benchmarks performed here, requiring at most four centroid structures to adequately describe the diversity of conformations in the sequences tested (Supplementary Table S2). Our approach avoids interpreting the SHAPE reactivities in the absence of structure models, which can lead to artifacts for methods that generate a single structure. For example, a nucleotide with moderate reactivity (e.g. a normalized reactivity of 0.75 on a scale of 0 to ∼2.5) might be unpaired, base paired, or in an ensemble of structures where it is paired in some structures (with reactivity of 0) and unpaired in other structures (with reactivity of 2). Each of these scenarios, including the number of states needed in the ensemble, is explicitly modeled by Rsample.

The modeling of structure populations relies on the thermodynamic nearest neighbor model for RNA folding (53) and the experimentally-derived restraints. Generally, the populations modeled by Rsample match the known populations to about a kcal/mol in free energy change. For example, the well characterized bistable sequence is populated at a ratio of 70–30 according to NMR (Table 2) (47), and is modeled by Rsample to be at a ratio of 94–6. At 310 K, this is a ΔΔ*G*° of 1.2 kcal/mol, or ∼2*kT*. These are relatively small differences in folding free energy change, although continued efforts to improve the thermodynamic models will continue to improve the agreement with experiments (54,55).

In our current implementation of Rsample, the per-nucleotide reactivities are estimated using three possible states: unpaired, paired at helix end, or paired at a helix interior. The modeling is flexible, however, and any functional form that depends on secondary structure could be used. For instance, we could increase from three states to additional states that could account for non-canonical base pairs that have flexible structures in specific sequence contexts, such as G–G or A–A pairs (56,57). As we learn more about what determines SHAPE reactivity, this functional form can be improved. A current weakness is that the distributions for the current three possible states (Figure 1c) are broad. For nucleotides that exist in one state in the initial ensemble, our estimate for reactivity tends towards the mean of the distribution. That means, for example, that high reactivity cannot be exactly matched by the model, and Rsample tends to restrain nucleotides with high experimental reactivity with less pairing tendency. Conversely, nucleotides with low experimental normalized reactivity (<0) tend to receive more propensity for being paired. Using a more complex functional form for per-nucleotide reactivity distributions, as mentioned above, could eventually help with this problem, as could the use of additional chemical probes alongside SHAPE. The current functional form of *ΔG_bonus, i_* (equation 1) is a heuristic that could also be refined as we learn more about what determines SHAPE reactivity.

The thermodynamic model for predicting RNA structure is central to Rsample. A single pass of refinement using the SHAPE data is sufficient for improving the secondary structure models. We observed that multiple passes (for example a loop in Figure 1A that connects step 6 back to step 3) degrades the accuracy performance. For example, if two passes of refinement are performed root-mean-square deviation between experimental and predicted reactivity decreased from 0.74 to 0.72 when comparing to the default one pass option, while the accuracy of prediction decreased from 82.8% to 71.0% for the single conformation dataset (see Supplementary Figure S5 in the Supplementary Data). It appears that multiple passes over-interpret the mapping data and take the ensemble farther from the thermodynamic model. The prediction is corrected in step 4 by adding restraints, allowing Rsample to perform well.

Although the goal of Rsample was to model multiple conformations using a principled approach, the performance on sequences with a single structure was not negatively affected (Figure 3). The difference in accuracy of Rsample compared to any of the tested methods that use experimental data was not statistically significant (as measured by a paired *t*-test). At the same time, on this dataset all methods perform about 10% better than predictions without experimental restraint data (Figure 3).

## AVAILABILITY

Rsample is a component of the RNAstructure software package, and is made available for free download using the GNU public license at our website: http://rna.urmc.rochester.edu/RNAstructure.html.

## Supplementary Material

Supplementary Data
