# Supplementary material for: Modeling RNA secondary structure folding ensembles using SHAPE mapping data
Source: Nucleic Acids Res. 2017 Nov 21;46(1):314–23. doi: 10.1093/nar/gkx1057 (PMC5758915; doi:10.1093/nar/gkx1057)
Supplement: Supplementary Data [file gkx1057_supp.pdf]

Supplementary Data to accompany

Modeling RNA Secondary Structure Folding Ensembles Using SHAPE Mapping Data

Aleksandar Spasic, Sarah M. Assmann, Philip C. Bevilacqua, & David H. Mathews

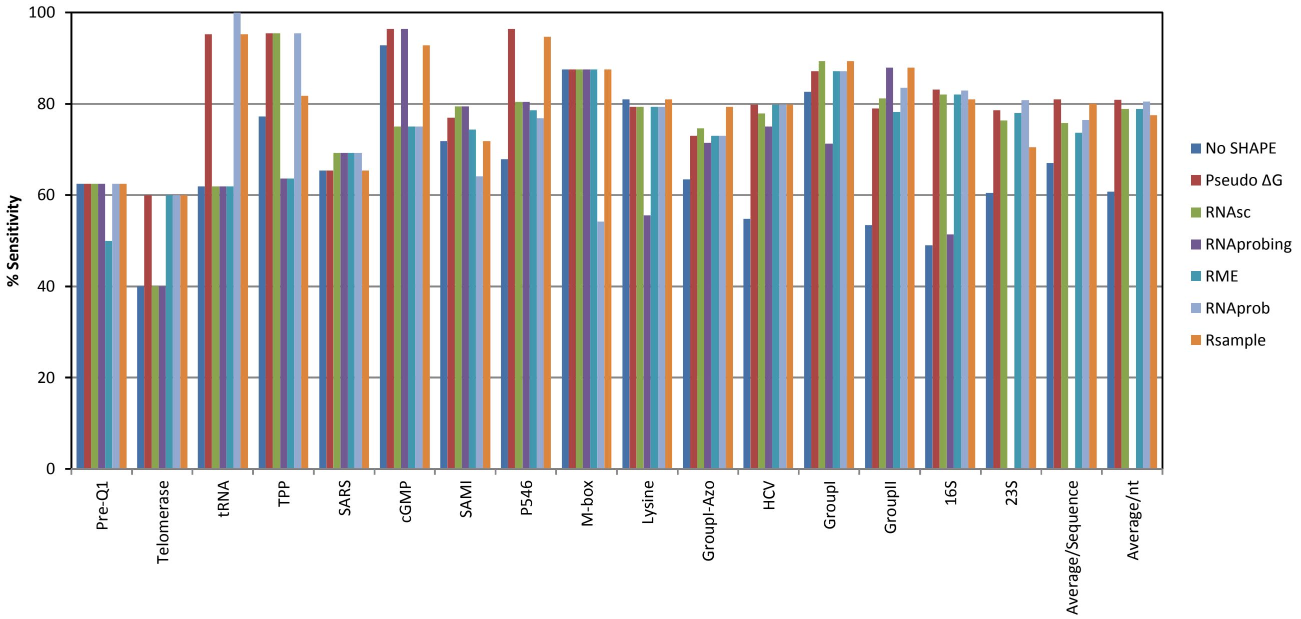

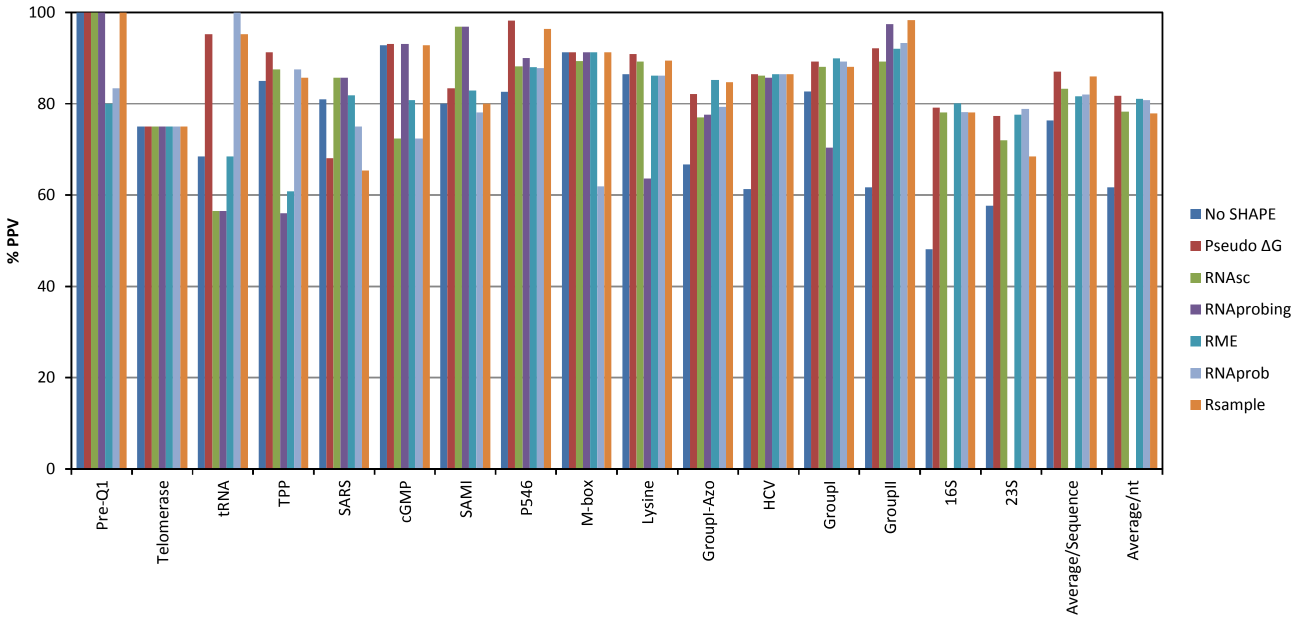

Figure S1. Performance of our method (Rsample) compared to other commonly used methods for predicting single conformation. The results are given as percent sensitivity and positive predictive values. RNAprobing predictions for 16S rRNA, 23S rRNA, and its average over all sequences and per nucleotide are left out because RNAprobing cannot handle sequences longer than 1,000 nucleotides.

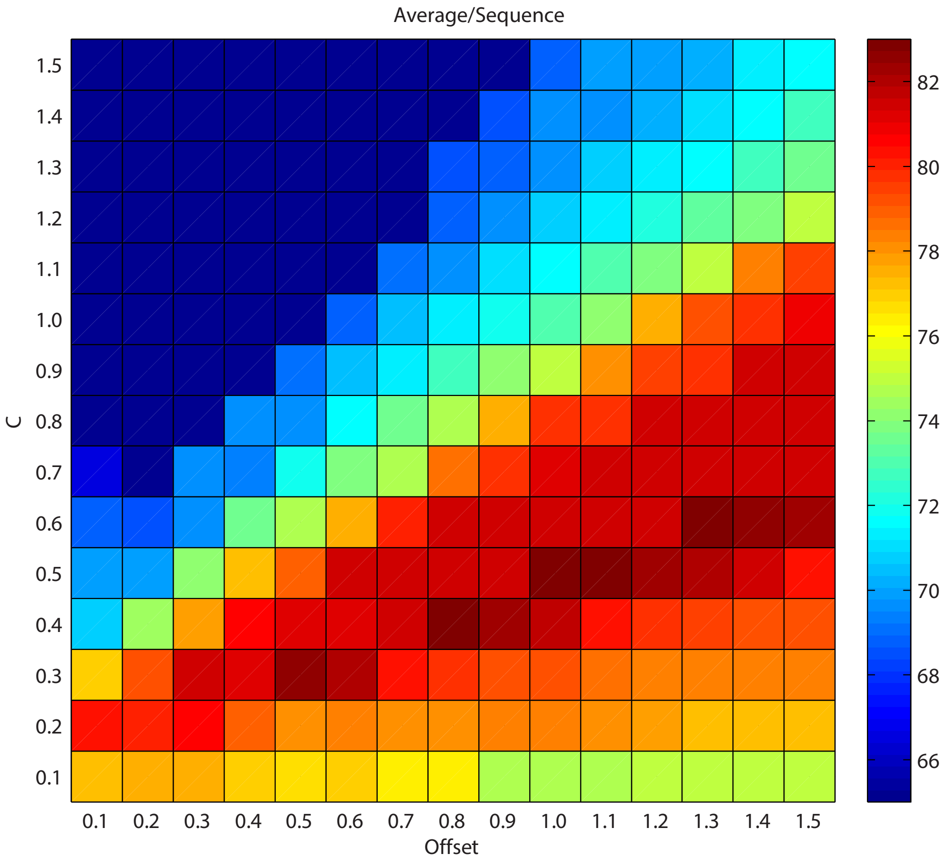

Figure S2. Geometric mean of sensitivity and positive predictive as a function of parameters *C* and *Offset*. Sensitivity and positive predictive value are arithmetic means of all 16 sequences from Table S1. The maximum value in this grid is 82.82% at *C*=0.5 kcal/mol and *Offset*=1.1. There is a broad range of values where average geometric mean of sensitivity and positive predictive value is larger than 80%.

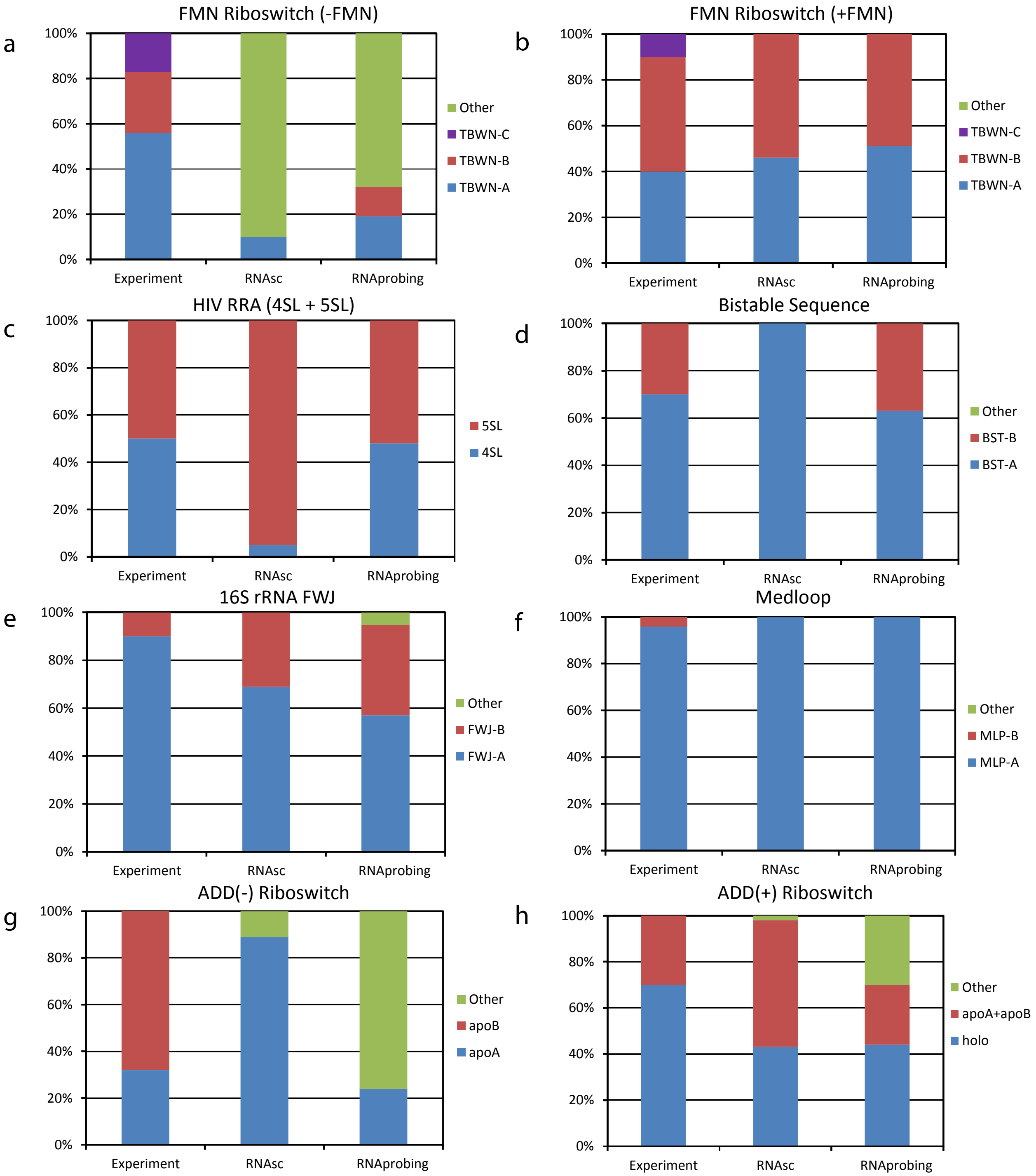

Figure S3. Experimental ratios of conformations are compared to the predicted ratios for the methods RNAprobing (1) and RNAsc (2). FMN riboswitch (-FMN; a), FMN riboswitch (+FMN, b), HIV RRA (c), bistable sequence (d), 16S rRNA FWJ (e), medloop (f), ADD riboswitch (-adenine; g), ADD riboswitch (+adenine; h). To illustrate the performance of the whole predicted ensemble, estimated populations for structures that were not close to any of the known conformations ("other") is also given as the green portion of the bar.

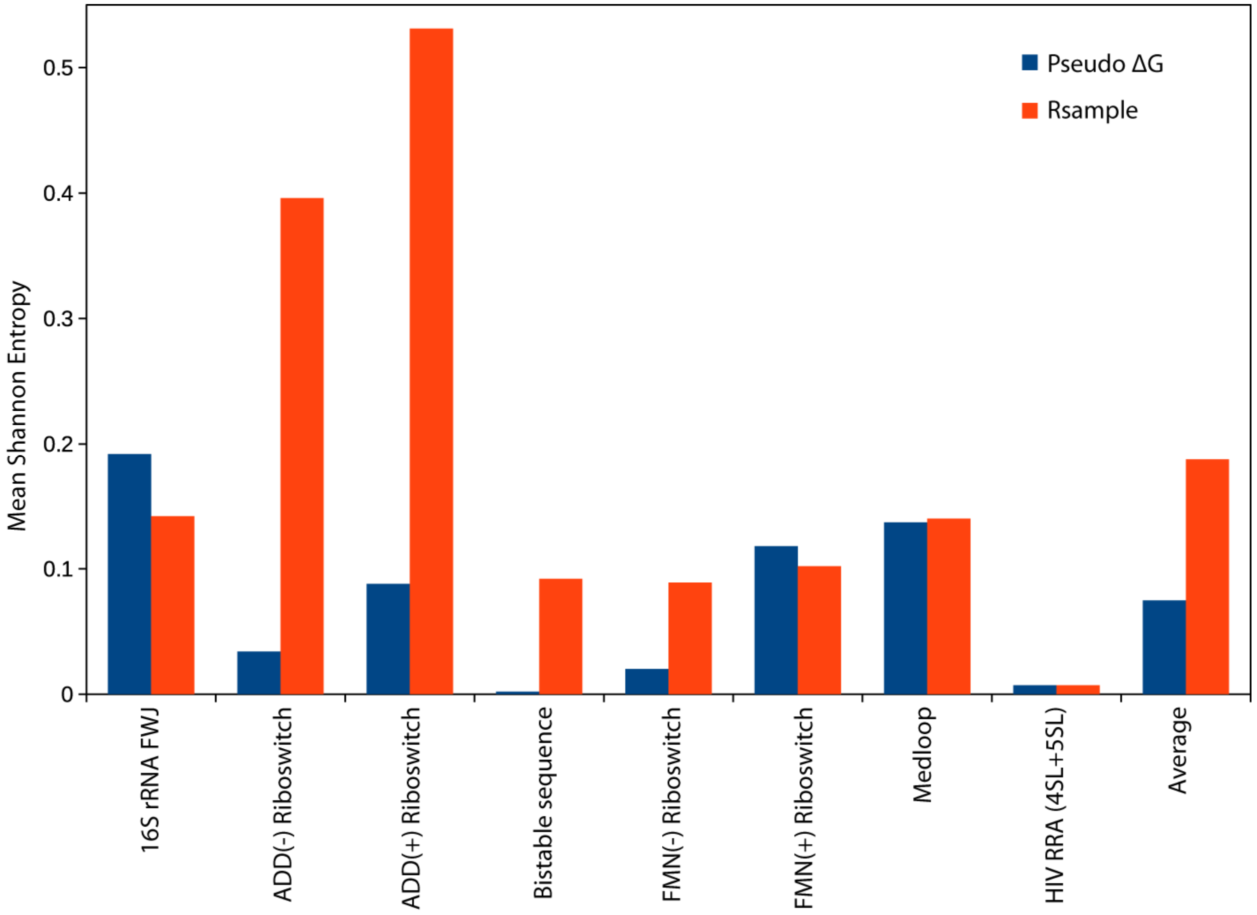

Figure S4. Mean Shannon entropy per nucleotide calculated for the sequences with multiple conformations (Table 1). Predictions from Rsample are in red, while the predictions using a previous method for including SHAPE restraints (pseudo ΔG) are in blue. The last column is the average over all sequences for the two methods.

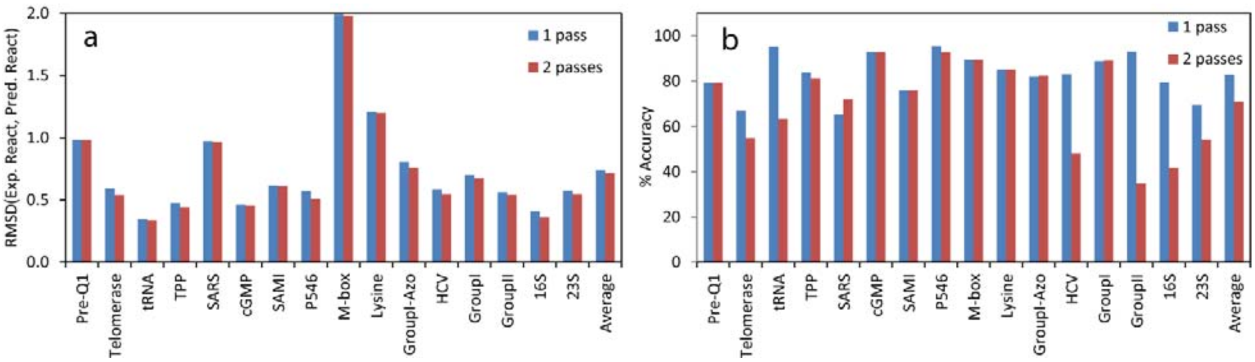

Figure S5. Performance of Rsample on the sequences from the single conformation dataset (Table S1) using one pass (the default option, blue bars) and two passes (when the prediction from step 6 in Figure 1a are used to re-estimate reactivities in step 3, red bars) methods. Panel a shows the root-mean-square deviation between experimental and predicted reactivities. Panel b shows the accuracy (geometric mean of sensitivity and PPV) of secondary structure prediction for one-pass and two-pass methods. The last columns in both panels are the averages over all sequences.

Table S1. List of sequences and their lengths used in calculating the distributions of unpaired, and paired in the middle and in the end nucleotides, calculating the parameters C and Offset and benchmarking the methods for single conformation prediction. Sequences, their secondary structures and their SHAPE data were taken from the dataset provided with the work of Hajdin et al. (3)

	Name	Length
1	Pre-Q1 riboswitch, *B. subtilis*	34
2	Telomerase, human	47
3	tRNA(asp), yeast	75
4	TPP riboswitch, *E. coli*	79
5	SARS, Corona virus	82
6	Cyclic-di-GMP riboswitch, *V. chloreae*	97
7	SAMI I riboswitch, *T. tengcongensis*	118
8	M-box riboswitch, *B. Subtilis*	154
9	P546 domain, bl3 group I intron	155
10	Lysine riboswitch, *T. maritama*	174
11	Group I intron, *Azoarcus Sp.*	214
12	Hepatitis C Virus (HCV) IRES domain	336
13	Group II intron, *O. iheyensis*	412
14	Group I intron, *T. thermophilia*	425
15	16S rRNA, *E. coli*	1542
16	23S rRNA, *E. coli*	2904

Table S2. Sensitivity and positive predictive value (PPV) of centroids of all clusters obtained using the 3 programs tested here (Left column). For each sequence (separate table), name (‘C1’, ‘C2’,…) and size of clusters (number of structures of a sample of 1000) is reported as well as the sensitivities and PPV of their centroids to the known conformations. The 50:50 mixture of 4SL and 5SL was generated by taking the mean SHAPE reactivity of the 4SL and 5SL SHAPE reactivities.

HIV-1 RRA:

			Sensitivity (left) and PPV (right) of centroid to conformation 1: 4 stem-loop (4SL)		Sensitivity (left) and PPV (right) of centroid to conformation 2: 5 stem-loop (5SL)	
No SHAPE		C1: 41	70.0	68.3	90.1	89.0
		C2: 882	80.0	78.1	100.0	98.8
		C3: 74	77.5	77.2	98.8	100.0
		C4: 3	100.0	100.0	77.8	78.8
Rsample	100% 4SL	C1: 281	78.8	77.8	100.0	100.0
		C2: 719	98.8	100.0	77.8	79.8
	100% 5SL	C1: 987	78.8	77.8	100.0	100.0
		C2: 13	100.0	100.0	77.8	78.8
	50% 4SL+ 50% 5SL	C1: 877	78.8	77.8	100.0	100.0
		C2: 123	98.8	100.0	77.8	80.0
Pseudo ΔG	100% 4SL	C1: 861	98.8	100.0	77.8	79.8
		C2: 139	78.8	79.8	97.5	100.0
	100% 5SL	C1: 857	78.8	78.8	98.8	100.0
		C2: 81	75.0	75.0	95.1	96.3
		C3: 62	77.5	78.2	97.5	100.0
	50% 4SL + 50% 5SL	C1: 931	78.8	78.8	98.8	100.0
		C2: 56	77.5	78.2	97.5	100.0
		C3: 10	82.5	83.5	93.8	96.2
		C4: 3	98.8	100.0	76.5	78.5

Bistable Hairpin:

		Sensitivity (left) and PPV (right) of centroid to conformation 1	Sensitivity (left) and PPV (right) of centroid to conformation 2
No SHAPE	C1: 727	100.0 100.0	0.0 0.0
	C2: 228	87.5 100.0	0.0 0.0
	C3: 9	87.5 100.0	0.0 0.0
	C4: 17	0.0 0.0	83.3 100.0
	C5: 8	75.0 100.0	0.0 0.0
	C6: 2	87.5 100.0	0.0 0.0
	C7: 1	87.5 100.0	0.0 0.0
	C8: 1	87.5 87.5	0.0 0.0
	C9: 6	62.5 100.0	0.0 0.0
	C10: 1	87.5 100.0	0.0 0.0
Rsample	C1: 887	100.0 100.0	0.0 0.0
	C2: 57	0.0 0.0	83.3 100.0
	C3: 56	75.0 100.0	0.0 0.0
Pseudo ΔG	C1: 154	75.0 100.0	0.0 0.0
	C2: 650	100.0 100.0	0.0 0.0
	C3: 63	87.5 100.0	0.0 0.0
	C4: 95	87.5 100.0	0.0 0.0
	C5: 11	87.5 100.0	0.0 0.0
	C6: 18	62.5 100.0	0.0 0.0
	C7: 2	75.0 100.0	0.0 0.0
	C8: 4	75.0 100.0	0.0 0.0
	C9: 1	87.5 100.0	0.0 0.0
	C10: 2	62.5 100.0	0.0 0.0

16S rRNA Four Way Junction (FWJ):

		Sensitivity (left) and PPV (right) of centroid to conformation 1		Sensitivity (left) and PPV (right) of centroid to conformation 2	
No SHAPE	C1: 83	31.4	31.4	41.7	42.9
	C2: 898	60.0	70.0	86.1	100.0
	C3: 19	42.9	53.6	63.9	82.1
Rsample	C1: 373	65.7	71.9	91.7	100.0
	C2: 609	97.1	100.0	63.9	69.7
	C3: 12	45.7	76.2	22.2	38.1
	C4: 6	22.9	34.8	30.6	43.5
Pseudo ΔG	C1: 688	60.0	77.8	75.0	100.0
	C2: 312	82.9	100.0	58.3	72.4

(FMN-) Riboswitch:

		Sensitivity (left) and PPV (right) of centroid to conformation 1	Sensitivity (left) and PPV (right) of centroid to conformation 2	Sensitivity (left) and PPV (right) of centroid to conformation 3
No SHAPE	C1: 566<br>C2: 434	85.7 100.0<br>0.0 0.0	0.0 0.0<br>0.0 0.0	0.0 0.0<br>0.0 0.0
Rsample	C1: 719<br>C2: 208<br>C3: 73	85.7 100.0<br>0.0 0.0<br>0.0 0.0	0.0 0.0<br>100.0 66.7<br>0.0 0.0	0.0 0.0<br>0.0 0.0<br>75.0 100.0
Pseudo ΔG	C1: 895<br>C2: 105	42.9 100.0<br>0.0 0.0	0.0 0.0<br>0.0 0.0	0.0 0.0<br>0.0 0.0

(FMN+) Riboswitch:

		Sensitivity (left) and PPV (right) of centroid to conformation 1	Sensitivity (left) and PPV (right) of centroid to conformation 2	Sensitivity (left) and PPV (right) of centroid to conformation 3
No SHAPE	C1: 566<br>C2: 434	85.7 100.0<br>0.0 0.0	0.0 0.0<br>100.0 61.5	0.0 0.0<br>0.0 0.0
Rsample	C1: 485<br>C2: 468<br>C3: 47	0.0 0.0<br>85.7 60.0<br>0.0 0.0	100.0 61.5<br>0.0 0.0<br>0.0 0.0	0.0 0.0<br>0.0 0.0<br>66.7 100.0
Pseudo ΔG	C1: 801<br>C2: 199	85.7 60.0<br>0.0 0.0	0.0 0.0<br>100.0 72.7	0.0 0.0<br>0.0 0.0

(ADD-) Riboswitch:

		Sensitivity (left) and PPV (right) of centroid to apoA		Sensitivity (left) and PPV (right) of centroid to apoB		Sensitivity (left) and PPV (right) of centroid to holo	
No SHAPE	C1: 303	64.3	54.6	70.4	57.6	48.0	36.4
	C2: 518	85.7	80.0	74.1	66.7	72.0	60.0
	C3: 19	42.9	41.4	51.9	48.3	24.0	20.7
	C4: 99	64.3	58.1	100.0	87.1	48.0	38.7
	C5: 43	64.3	60.0	74.1	66.7	48.0	40.0
Rsample	C1: 887	85.7	80.0	74.1	66.1	72.0	60.0
	C2: 113	57.1	80.0	22.2	30.0	76.0	95.0
Pseudo ΔG	C1: 132	78.6	88.0	70.4	76.0	72.0	72.0
	C2: 497	78.6	84.6	74.1	76.9	72.0	69.2
	C3: 291	78.6	95.7	63.0	73.9	72.0	78.3
	C4: 18	75.0	83.3	70.4	79.2	68.0	66.7
	C5: 32	78.6	81.5	74.1	74.1	72.0	66.7
	C6: 11	78.6	91.7	63.0	70.1	72.0	75.0
	C7: 16	85.7	96.0	63.0	68.0	80.0	80.0

(ADD+) Riboswitch:

		Sensitivity (left) and PPV (right) of centroid to apoA		Sensitivity (left) and PPV (right) of centroid to apoB		Sensitivity (left) and PPV (right) of centroid to holo	
No SHAPE	C1: 303	64.3	54.6	70.4	57.6	48.0	36.4
	C2: 518	85.7	80.0	74.1	66.7	72.0	60.0
	C3: 19	42.9	41.4	51.9	48.3	24.0	20.7
	C4: 99	64.3	58.1	100.0	87.1	48.0	38.7
	C5: 43	64.3	60.0	74.1	66.7	48.0	40.0
Rsample	C1: 498	64.3	75.0	74.1	83.3	48.0	50.0
	C2: 386	57.1	80.0	22.2	30.0	76.0	95.0
	C3: 82	39.3	42.3	44.4	46.2	24.0	23.0
	C4: 34	39.3	45.8	22.2	25.0	48.0	50.0
Pseudo ΔG	C1: 155	85.7	89.0	63.0	63.0	88.0	81.5
	C2: 844	78.6	84.6	44.4	46.2	100.0	96.2

MedLoop sequence:

		Sensitivity (left) and PPV (right) of centroid to conformation 1	Sensitivity (left) and PPV (right) of centroid to conformation 2
No SHAPE	C1: 322<br>C2: 322<br>C3: 353	100.0 100.0<br>100.0 71.4<br>100.0 83.3	0.0 0.0<br>0.0 0.0<br>0.0 0.0
Rsample	C1: 548<br>C2: 444<br>C3: 8	100.0 83.3<br>100.0 71.4<br>0.0 0.0	0.0 0.0<br>0.0 0.0<br>100.0 90.0
Pseudo ΔG	C1: 39<br>C2: 391<br>C3: 416<br>C4: 59<br>C5: 87<br>C6: 8	100.0 100.0<br>100.0 71.4<br>100.0 83.3<br>100.0 71.4<br>100.0 71.4<br>100.0 83.3	0.0 0.0<br>0.0 0.0<br>0.0 0.0<br>0.0 0.0<br>0.0 0.0<br>0.0 0.0

Table S3. Accuracy of RNAprobing (1) and RNAsc (2) for predicting the structures of sequences with multiple conformations. The reported quantity is the accuracy, i.e. geometric mean of sensitivity and positive predictive value in percent (where 100 is a perfect prediction). We report up to 3 largest clusters for clarity. The sizes of clusters from which centroids were derived are reported in parenthesis as the percent of the total sampled ensemble. Marked in red are correctly identified conformations.

		RNAsc			RNAprobing		
20 Experimental Conformations:		C1	C2	C3	C1	C2	C3
Bistable sequence		(89)	(10)		(63)	(37)	
	Conf. 1	86.6	79.1		100	0	
	Conf. 2	0	0		0	100	
16S rRNA FWJ		(69)	(31)		(57)	(38)	(5)
	Conf. 1	98.6	72.2		94.1	64.2	18.8
	Conf. 2	65.7	89.8		62.9	83.3	34.0
HIV-1 RRA		(95)	(5)		(52)	(48)	
	4SL	79.3	98.8		68.3	99.4	
	5SL	98.8	78.3		90.1	78.8	
ADD(-) Riboswitch		(84)	(10)	(5)	(24)	(20)	(15)
	ApoA	85.2	66.8	78.5	79.4	25.4	26.0
	ApoB	67.9	59.0	73.9	73.1	25.8	26.5
	Holo	66.7	51.8	57.5	68.0	26.8	27.5
ADD(+) Riboswitch		(31)	(25)	(23)	(44)	(26)	(19)
	ApoA	82.1	91.2	81.5	67.6	78.6	19.5
	ApoB	69.1	58.2	41.5	25.8	72.4	0
	Holo	64.2	81.7	94.1	85.0	68.0	31.0
MedLoop sequence		(65)	(23)	(8)	(47)	(23)	(17)
	Conf. 1	81.7	87.7	84.5	84.5	100	91.3
	Conf. 2	0	0	0	0	0	0
FMN(-) Riboswitch		(58)	(28)	(10)	(68)	(19)	(13)
	Conf. 1	0	65.5	84.5	0	92.6	0
	Conf. 2	0	0	0	0	0	94.3
	Conf. 3	0	0	0	0	0	0
FMN(+) Riboswitch		(54)	(46)		(51)	(49)	
	Conf. 1	0	71.7		87.5	0	
	Conf. 2	80.2	0		0	94.3	
	Conf. 3	0	0		0	0	

References:

1. Washietl, S., Hofacker, I.L., Stadler, P.F. and Kellis, M. (2012) RNA folding with soft constraints: reconciliation of probing data and thermodynamic secondary structure prediction. *Nucleic Acids Res.*, **40**, 4261-4272.
2. Zarringhalam, K., Meyer, M.M., Dotu, I., Chuang, J.H. and Clote, P. (2012) Integrating chemical footprinting data into RNA secondary structure prediction. *PLoS One*, **7**, e45160.
3. Hajdin, C.E., Bellaousov, S., Huggins, W., Leonard, C.W., Mathews, D.H. and Weeks, K.M. (2013) Accurate SHAPE-directed RNA secondary structure modeling, including pseudoknots. *Proc. Natl. Acad. Sci. U.S.A.*, **110**, 5498-5503.
